# Who Benefits in Vitality After Retirement? Findings From a 3-Year Panel Study

**DOI:** 10.1093/geroni/igaa057.1502

**Published:** 2020-12-16

**Authors:** Anushiya Vanajan, Ute Bültmann, Kène Henkens

**Affiliations:** 1 Netherlands Interdisciplinary Demographic Institute, Den Haag, Netherlands; 2 University of Groningen, Groningen, Netherlands; 3 NIDI, Den Haag, Netherlands

## Abstract

Background. Past studies have revealed the effect of retirement on various health measures. None, however, have studied retirement’s effect on vitality, a holistic measure of physical and mental health. To boot, very few studies have addressed the heterogeneity in the health consequences of retirement. This study investigates the effect of retirement on vitality, and how this effect is influenced by 1) manual work and 2) baseline vitality. Methods. The analyses were based on two waves of the NIDI Pension Panel Survey, collected in the Netherlands in 2015 and 2018. Data from 4,156 older workers (N=4,156), of whom 1,934 (46.5%) retired between waves, were analysed. Vitality is assessed in three ways, as: 1) a composite measure of vitality, and its sub-components 2) energy and 3) fatigue. Results. Conditional Change OLS Regression Models demonstrate that retirement improves vitality and decreases fatigue. These effects were heterogeneous. Retirement was more advantageous for older workers who experienced poor vitality and increased fatigue before retirement. Likewise, older workers who were employed in manual work before retirement, experienced the largest gains in vitality and deepest declines in fatigue post-retirement. No such effects were found for energy. Conclusions. Older workers experiencing low baseline vitality and high baseline fatigue and those in manual labor may benefit from early retirement. Since early retirement is financially unfavorable, it is essential to provide these groups of workers with workplace vitality interventions that may not only improve their vitality and quality of working life, but also extend their participation in the labor market.

